# Ambient Ultrafine Particle Ingestion Alters Gut Microbiota in Association with Increased Atherogenic Lipid Metabolites

**DOI:** 10.1038/srep42906

**Published:** 2017-02-17

**Authors:** Rongsong Li, Jieping Yang, Arian Saffari, Jonathan Jacobs, Kyung In Baek, Greg Hough, Muriel H. Larauche, Jianguo Ma, Nelson Jen, Nabila Moussaoui, Bill Zhou, Hanul Kang, Srinivasa Reddy, Susanne M. Henning, Matthew J. Campen, Joseph Pisegna, Zhaoping Li, Alan M. Fogelman, Constantinos Sioutas, Mohamad Navab, Tzung K. Hsiai

**Affiliations:** 1 Division of Cardiology, Department of Medicine, School of Medicine, University of California, Los Angeles, CA 90095, USA; 2 Division of Clinical Nutrition, Department of Medicine, School of Medicine, University of California, Los Angeles, CA 90095, USA; 3 Civil and Environmental Engineering, University of Southern California, Los Angeles, CA 90089, USA; 4 Division of Gastroenterology and Hepatology, Department of Medicine, School of Medicine, University of California, Los Angeles, CA 90095, USA; 5 Department of Bioengineering, School of Engineering & Applied Science, University of California, Los Angeles, CA 90095, USA; 6 Department of Pharmaceutical Sciences, School of Pharmacy, University of New Mexico, Albuquerque, NM 87131, USA

## Abstract

Ambient particulate matter (PM) exposure is associated with atherosclerosis and inflammatory bowel disease. Ultrafine particles (UFP, *d*_p_ < 0.1–0.2 μm) are redox active components of PM. We hypothesized that orally ingested UFP promoted atherogenic lipid metabolites in both the intestine and plasma via altered gut microbiota composition. Low density lipoprotein receptor-null (*Ldlr*^−/−^) mice on a high-fat diet were orally administered with vehicle control or UFP (40 μg/mouse/day) for 3 days a week. After 10 weeks, UFP ingested mice developed macrophage and neutrophil infiltration in the intestinal villi, accompanied by elevated cholesterol but reduced coprostanol levels in the cecum, as well as elevated atherogenic lysophosphatidylcholine (LPC 18:1) and lysophosphatidic acids (LPAs) in the intestine and plasma. At the phylum level, Principle Component Analysis revealed significant segregation of microbiota compositions which was validated by Beta diversity analysis. UFP-exposed mice developed increased abundance in Verrocomicrobia but decreased Actinobacteria, Cyanobacteria, and Firmicutes as well as a reduced diversity in microbiome. Spearman’s analysis negatively correlated Actinobacteria with cecal cholesterol, intestinal and plasma LPC18:1, and Firmicutes and Cyanobacteria with plasma LPC 18:1. Thus, ultrafine particles ingestion alters gut microbiota composition, accompanied by increased atherogenic lipid metabolites. These findings implicate the gut-vascular axis in a atherosclerosis model.

Ultrafine particles (UFP, *d*_p_ < 0.1–0.2 μm) are redox active components of airborne particulate matter (PM) that are enriched in transition metals and cycling organic chemicals^1^,^2^. In addition to inducing oxidative stress in human aortic endothelial cells^3^, UFP exposure reduces anti-oxidant capacity of plasma high-density lipoprotein (HDL) and increases oxidative lipid metabolism to accelerate atherosclerosis in low-density lipoprotein (LDL) receptor-knockout (*Ldlr*^−/−^) mice^4^,^5^. Recent epidemiological studies have linked ambient PM exposure with an increased risk for inflammatory bowel disease (IBD)^6^, and carotid intima thickness has also linked IBD with an increased risk for the development of atherosclerosis^7^. A large fraction of inhaled PM is recognized to be eliminated to the GI tract^8^,^9^. However, it remains elusive whether oral UFP ingestion induces intestinal inflammation to initiate atherosclerosis.

While the respiratory system is considered the primary route of inhaled PM exposure, the intestine is exposed to inhaled PM via mucociliary transport from the lungs to the gastrointestinal (GI) tract^10^,^11^. In the modern Western diet, more than 10^12^ UFP are orally ingested daily per person^12^. These ingested dietary UFP, including titanium oxide (TiO_2_) as whitening additives and alumino-silicates in drinking water, are absorbed by the intestinal intraepithelial lymphocytes that release pro-inflammatory cytokines to stimulate T-cell proliferation^11^,^13^,^14^,^15^,^16^. In addition to reducing the anti-oxidant capacity of HDL, UFP exposure induced intestinal release of pro-inflammatory mediators and fatty acid metabolites in *Ldlr*-null mice^4^,^5^. Urban coarse particulate matter (PM_10_, *d* < 10 μm) ingested via contaminated food altered gut microbiota in IL-10-null mice, an IBD mouse model^17^. For this reason, we sought to study the role of UFP ingestion on gut microbiota in *Ldlr*-null mice to alter lipid metabolism and atherogenic lipid metabolites.

The gut of human and many other vertebrates is mostly dominated by two phyla of bacteria, Bacteroidetes and Firmicutes^18^. Minor populations of Actinobacteria, Fusobacteria, and Cyanobacteria species are also present, as part of a complex microbial community^18^. Dysbiosis, an imbalance in the gut microbiota, may modulate host metabolism, immunity, and inflammatory responses resulting in pathological conditions. Mounting evidence has supported the link between the intestinal microbiome and human diseases, including cardiovascular, gastrointestinal, metabolic, neurological diseases, cancer, and obesity^19^,^20^,^21^,^22^,^23^,^24^,^25^,^26^,^27^. Gut microbiome-induced changes in lipid metabolism are associated with intestinal inflammation^20^,^23^,^28^,^29^ and gut microbiota-dependent formation of dietary trimethylamine (TMA) is linked with atherosclerosis^25^,^30^.

In this context, building on our previous inhalation study in which UFP exposure promoted inflammatory responses and lipid metabolism in both the gastrointestinal and vascular systems^4^,^5^, we hereby tested the hypothesis that oral UFP ingestion altered gut microbiota to promote intestinal and serum pro-inflammatory mediators and atherogenic lipid metabolites in *Ldlr*-null mice. Our findings suggest gut-vascular transmissibility via UFP-mediated changes in microbiome in a *Ldlr*-null mouse model of atherosclerosis.

## Results

### UFP ingestion segregated gut microbiota

**Figure 1:**
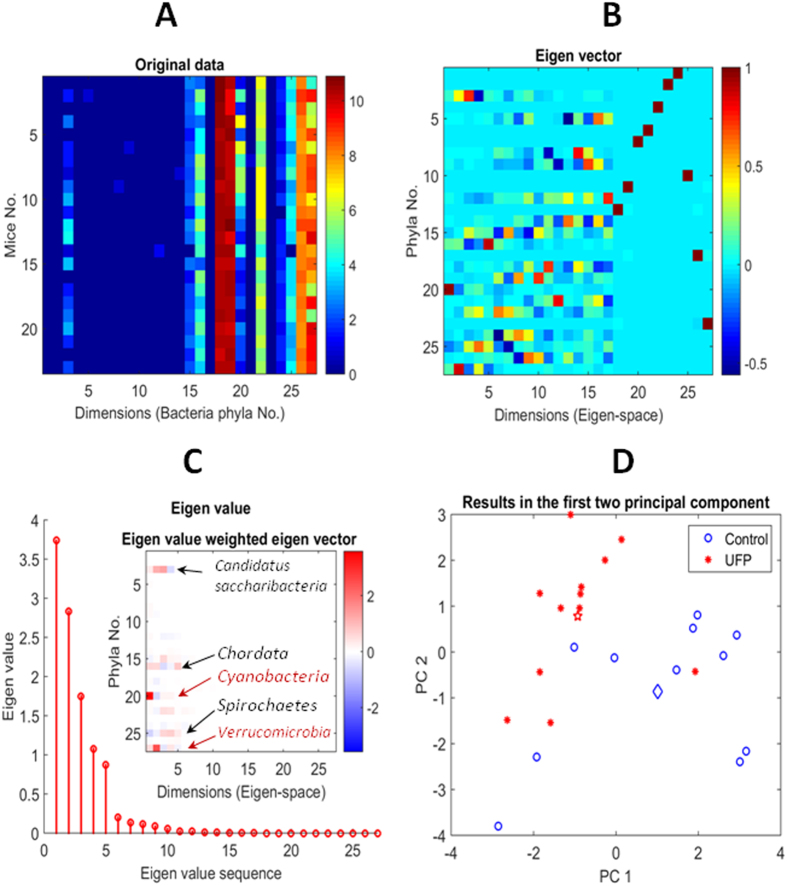
Principle Component Analysis (PCA) of Microbiota. DNAs were isolated from cecal contents for miseq sequencing. PCA was conducted with microbiota data from vehicle control (n = 11) and UFP-gavaged mice (n = 12). (**A**) The abundance (natural logarithm value) of each bacterial phylum (27 phyla) for the individual mice. (**B**) The characteristic pattern exhibited from Eigen vectors was calculated from the abundance data. (**C**) Eigen values calculated from abundance data indicated the main variance was from *Verrucomicrobia, Spirochaetes, Cyanobacteria, Chordata* and *Candidatus Saccharibacteria* as mapped in the inset. (**D**) In the principal component (first two Eigen vectors) space, the control and UFP groups exhibited distinct characteristics. The mean UFP value (red pentagram) is significantly lower than that of the control (blue diamond) in the first principal component space (PC1, *p* < 0.001).

We analyzed the gut microbial composition in the UFP-ingested *Ldlr*-null mice by isolating DNA from cecum contents, followed by MiSeq 16S ribosomal RNA gene sequencing to characterize the microbiome. The relative abundance of bacteria (Fig. 1A) was calculated at the phylum level. Principal Component Analysis (PCA) revealed segregation of microbiota between the control and UFP-ingested groups. The Eigen vectors and values calculated from phylum level abundance revealed that Candidatus Saccharibacteria (TM7), Cyanobacteria, Chordata, Verrucomicrobia, and Spirochaetes were significantly different in the UFP-ingested group (Fig. 1B,C). In the first Principal Component (PC1), the mean value (red pentagram) of the UFP-ingested group was significantly lower than that of the control group (blue diamond) (*p* < 0.001, n = 11–12) (Fig. 1D).

**Figure 2:**
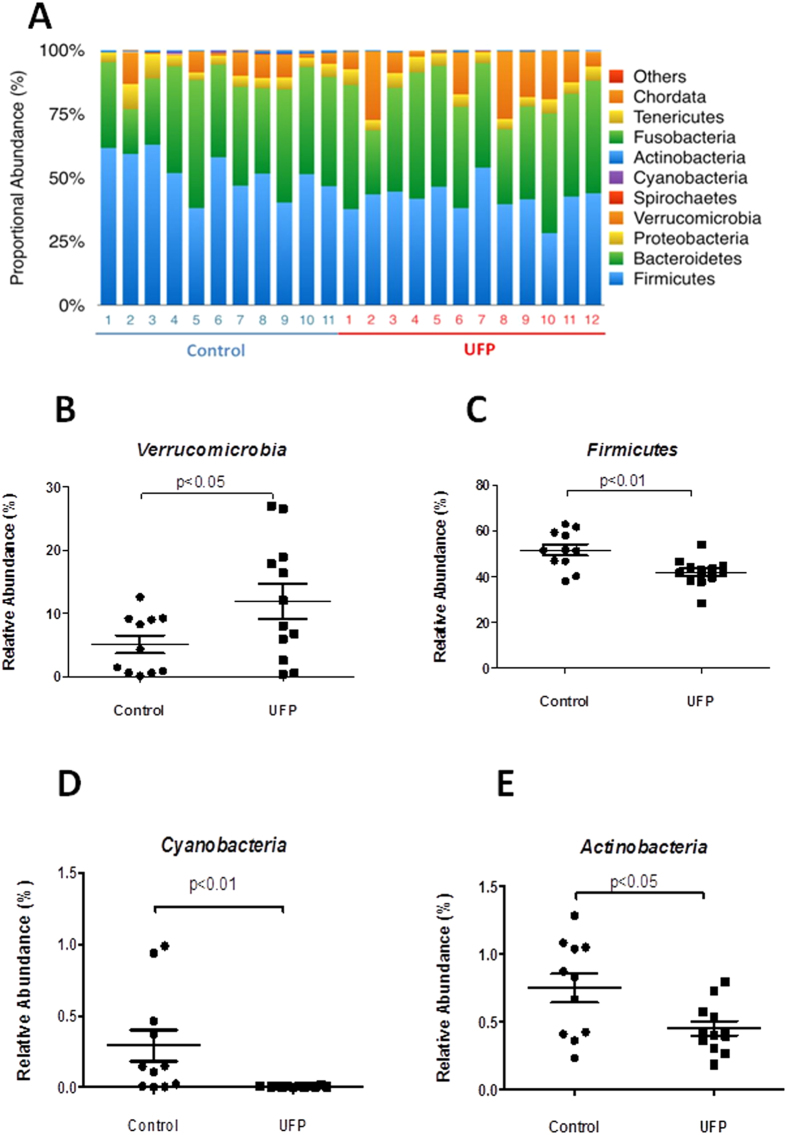
Gavaged UFP Altered cecal microbiota. DNAs were isolated from cecal contents for miseq sequencing. The relative abundance of bacteria was calculated based on operational taxonomic units (OTUs). (**A**) Overview of the relative abundance of gut bacteria depicted at the phylum level in mice exposed to vehicle control vs. UFP. (**B–E**) Relative abundance of *Verrocomicrobia, Firmicutes, Cyanobacteria,* and *Actinobacteria* was plotted against the control (n = 11–12).

UFP ingestion altered the relative abundance of 4 out of the 27 phyla (Fig. 2A). The relative abundance of Verrucomicrobia (associated with intestinal mucus degradation) was increased by 133.4 ± 52.9% (n = 11–12, *p* < 0.05) (Fig. 2B), Firmicutes was decreased by 19.0 ± 3.5% (n = 11–12, *p* < 0.01) (Fig. 2C), Cyanobacteria was reduced by 97.7 ± 0.4% (n = 11–12, *p* < 0.05) (Fig. 2D) and Actinobacteria was lowered by 40.1 ± 6.9% (n = 11–12, *p* < 0.05) (Fig. 2E). Actinobacteria, Cyanobacteria, and Firmicutes are recognized to associate with fatty acid absorption and lipid metabolism in mice^18^.

At the species level, PCA revealed significant segregation between the UFP-ingested and the control groups (PC1, *p < *0.0001) (Supplemental Figure S1A). UFP ingestion significantly altered the relative abundance of 54 out of 675 operational taxonomic units (OTUs), which roughly correspond to species (Supplemental Figure S1B). The relative abundance of *Akkermansia muciniphila,* the dominant species of Verrucomicrobia (Supplemental Figure S1C), was increased by ~2.4-fold, and two Lachnospiraceae species, including *Lachnoclostridium Clostridium saccharolyticum* and *Clostridium scindens,* were decreased by ~10-fold and ~2.2-fold, respectively (Supplemental Figure S1D,E). The Lachnospiraceae family as a whole (negatively associated with colon cancer) was reduced by ~1.8-fold (Supplemental Figure S1F). PCA also revealed that the UFP-ingested group had statistically significant different microbiome at the Class, Order, Family and Genus levels (*p* < 0.0001).

To verify the UFP-mediated distinct microbial compositions, we re-analyzed the data using an independent bioinformatics pipeline. Alpha diversity analysis revealed that UFP treated mice had a less diverse microbiome as assessed by Chao1, Faith’s phylogenetic diversity, and Shannon index (Supplemental Figure S2A). Beta diversity analysis using unweighted and weighted UniFrac further demonstrated statistically significant separation of microbial profiles between control and UFP-treated mice as assessed by a permutation-based method (Supplemental Figure S2B). Differential abundance testing using negative binomial models for OTU level data revealed that 29 OTUs were enriched and 53 were depleted in the UFP treated mice compared to control at a false discovery rate (FDR) threshold of 0.1 (Supplemental Figure S2C). This included five enriched OTUs identified as *Akkermansia muciniphila*, three depleted Cyanobacteria OTUs in the order YS2 and one depleted Actinobacteria OTU in the *Bifidobacterium* genus. Analysis at the phylum level confirmed enrichment of Verrucomicrobia and depletion of Cyanobacteria and Actinobacteria at a FDR threshold of 0.1.

### UFP ingestion increased intestinal inflammatory cells and plasma cytokines

**Figure 3:**
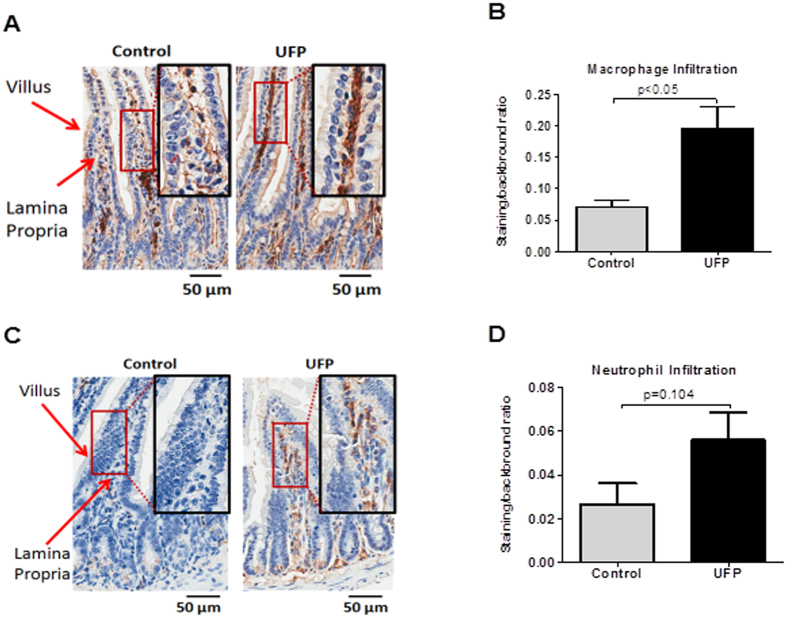
UFP ingestion promoted intestinal inflammation. Cross sections of ileum from mice exposed to vehicle control or UFP were stained with antibody F4/80 for macrophages and antibody against Ly6G for neutrophil. (**A**) Representative macrophage staining in the villi of ileum. (**B**) The averaged staining intensity of macrophages. (**C**) Representative neutrophil staining in the villi of ileum. (**D**) The averaged staining intensity of neutrophil. UFP ingestion significantly increased macrophage staining and exhibited a trend toward an increase in neutrophil stainnig (n = 7–11).

**Figure 4:**
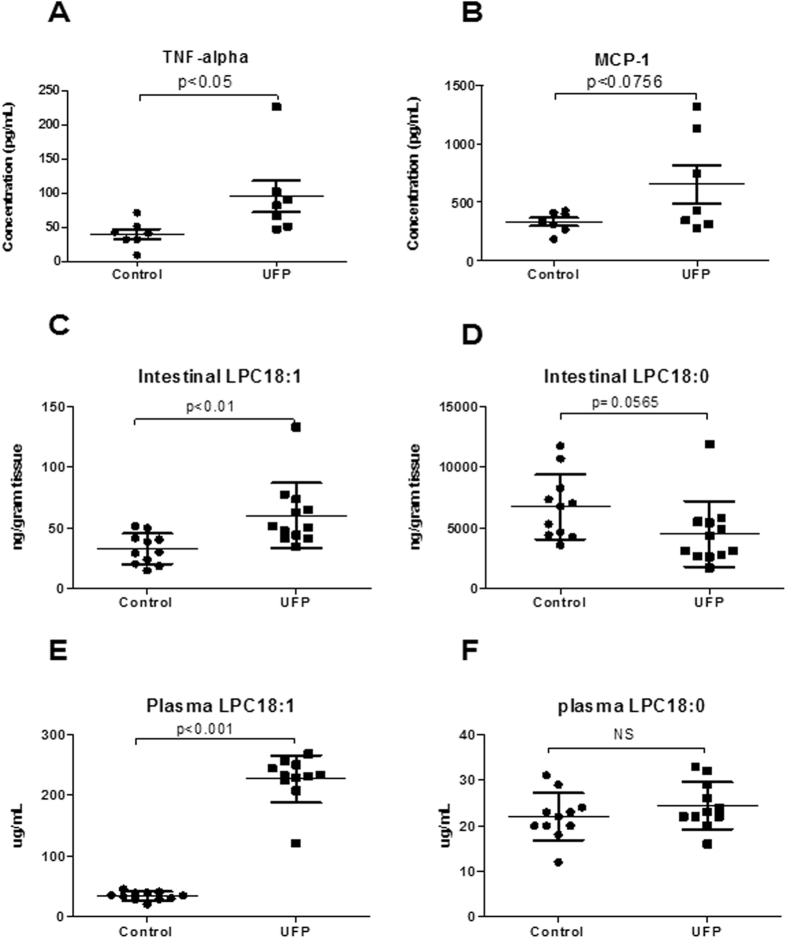
Gavaged UFP increased pro-inflammatory cytokines and lipid metabolites. Plasma levels of cytokine were measured by Luminex assay and the levels of lipid metabolites by LC-ESI-MS-MS. (**A**) Plasma TNF-α (n = 7). (**B**) Plasma MCP-1 (n = 7). (**C**) Intestinal LPC18:1 (n = 11–12). (**D**) Intestinal LPC18:0 (n = 11–12). (**E**) Plasma LPC18:1 (n = 11). (**F**) Plasma LPC18:0 (n = 11). Gavaged UFP significantly increased plasma TNF-α and LPC18:1 levels in both intestinal and plasma, and a trend of increase in MCP-1 (*p* = 0.0756, n = 7), whereas LPC 18:0 level was unchanged (n = 11–12).

Orally gavaged UFP increased F4/80 staining for macrophages by 3-fold (Control = 0.07 ± 0.01 vs. UFP = 0.2 ± 0.04, n = 8–11, *p* < 0.05) and anti-Ly6G staining for neutrophils by 2-fold (Control = 0.027 ± 0.0095 vs. UFP = 0.056 ± 0.0128, n = 8–11, *p* = 0.1) in the intestinal villi, though only the change in macrophages reached significance (Fig. 3). In parallel, both TNF-α and MCP-1 levels were elevated by 2-fold in the plasma, though the change in MCP-1 fell short of significance (TNF-α: Control = 40.3 ± 7.3, UFP = 95.2 ± 23.1 pg/mL, n = 7, *p* < 0.05; MCP-1: Control = 334.2 ± 33.3, UFP = 653.7 ± 160.9 pg/mL, n = 7, *p* = 0.08) (Fig. 4A,B).

Mass spectrometry analysis revealed elevated pro-atherogenic lipid metabolites, including lysophosphatidylcholine (LPC), lysophosphatidic acid (LPA), 1-palmitoyl-2-epoxyisoprostane E2-*sn*-glycero-3-phosphorylcholine (PEIPC), 1-palmitoyl-2-oxovaleroyl-*sn*-glycero-3-phosphorylcholine (POVPC) and 1-palmitoyl-2-glutaroyl-*sn*-glycero-3-phosphorylcholine (PGPC)^31^,^32^,^33^,^34^. LPC 18:1 was elevated by 1.8 fold in the intestine and ~6.5-fold in the plasma (intestine: Control = 32.8 ± 3.8, UFP = 60.2 ± 7.7 μg/g tissue, n = 11–12, *p* < 0.01; plasma: Control = 34.7 ± 2.1, UFP = 227.0 ± 11.7 μg/mL, n = 11, *p* < 0.001) (Fig. 4C,E), whereas LPC 18:0 remained unchanged (Fig. 4D,F). Furthermore, LPAs, including LPA18:1, LPA18:2 and LPA20:4, and oxidized phospholipids (PEIPC, POVPC, and PGPC) were significantly increased in the intestine and plasma (Supplemental Material Table S2). Hence, UFP ingestion promoted inflammatory responses in the intestine and increased various lipid metabolites, including the atherogenic LPC 18:1, in both the intestine and plasma.

### UFP ingestion decreased cholesterol metabolism in cecum

**Figure 5:**
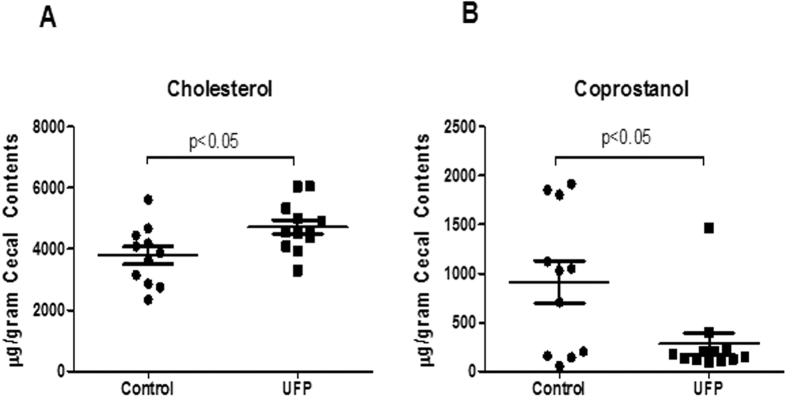
UFP ingestion modulated cecal cholesterol and its metabolites. Lipids from cecal contents were extracted by ethanol as described in methods. Cholesterol and cholesterol metabolite coprostanol were measured by gas chromatography. (**A**) Cecal cholesterol was elevated and (**B**) Cecal coprostanol was reduced by UFP ingestion (n = 11–12).

Cecal bile acids, cholesterol and its metabolite, coprostanol, were quantified. While there were no significant changes in the plasma cholesterol (Supplemental Figure S3) or cecal bile acids (Supplemental Material Table S3), cecal cholesterol was increased by 1.25-fold (Control = 3783.3 ± 289.9, UFP = 4715.3 ± 234.7 μg/gram cecal contents, n = 11–12, *p* < 0.05), whereas the cholesterol metabolite coprostanol was significantly reduced by 69% (Control = 912.2 ± 217.3, UFP = 283.7 ± 109.5 μg/g cecal contents, n = 11–12, *p* < 0.05) (Fig. 5).

### Association between UFP-altered microbiota and lipid metabolites

**Figure 6:**
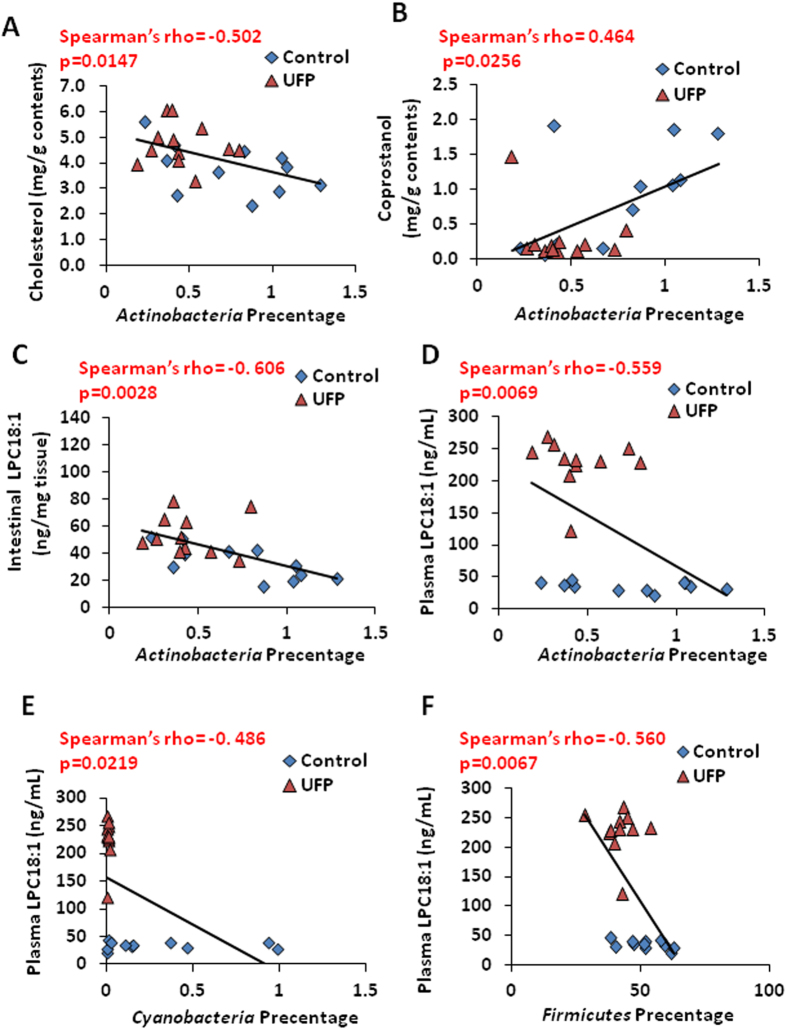
Correlation between microbiota and pro-inflammatory mediators. (**A**) The abundance of *Actinobacteria* was inversely correlated with cecal cholesterol. (**B**) *Actinobacteria* was positively correlated with coprostanol. (**C**) *Actinobacteria* was inversely correlated with intestinal LPC18:1. (**D**) *Actinobacteria* was inversely correlated with plasma LPC18:1. (**E**) Plasma LPC18:1 was negatively associated with *Cyanobacteria*. (**F**) Plasma LPC18:1 was negatively associated with *Firmicutes* (n = 11–12).

Spearman’s correlation analyses revealed that Actinobacteria was inversely correlated with intestinal cholesterol (ρ = −0.502, *p* < 0.05) (Fig. 6A), but positively correlated with coprostanol (ρ = 0.464, *p* < 0.05) (Fig. 6B). Actinobacteria (ρ = −0.559, *p* < 0.01), Cyanobacteria (ρ = −0.486, *p* < 0.05), and Firmicutes (ρ = −0.560, *p* < 0.01) were inversely correlated with plasma LPC 18:1 (Fig. 6C–F). Taken together, UFP-mediated reductions in Actinobacteria, Cyanobacteria, and Firmicutes are associated with pro-inflammatory cytokines and atherogenic lipid metabolites.

## Discussion

We demonstrate that chronic oral UFP ingestion to *Ldlr*-null mice engendered dysbiosis, including altered microbial composition and diversity in association with increased TNF-α and atherogenic LPC 18:1 and LPAs in the intestinal and plasma. Chronic UFP ingestion reduced the abundance of Lachnospiraceae (negatively associated with colon cancer)^35^,^36^,^37^ and increased Verrucomicrobia (intestinal mucus degradation)^38^,^39^ in the cecum. The reduction in Actinobacteria, Cyanobacteria, and Firmicutes was correlated with increased cecal cholesterol, and both intestinal and plasma levels of the lipid metabolite LPC 18:1. Thus, our finding provides new insight into gut-vascular transmissibility to initiate atherosclerosis via UFP-mediated segregation in microbiota.

Gut microbiota composition varies in natural populations and is influenced by environmental factors that regulate host metabolism, immunity, and inflammatory responses^20^,^23^,^24^,^25^,^26^,^27^. In our atherosclerosis model (UFP-ingested *Ldlr*-null mice), we observed an increased abundance in Verrucomicrobia (implicated in intestinal mucus degradation^39^) and a decreased abundance in Firmicutes (Fig. 2C), with a trend toward increased Bacteroidetes (data not shown). These findings are concordant with those of Kish *et al*., who reported an increase in the abundance of Verrucomicrobia and Firmicutes, but a decrease in Bacteroidetes, in PM_10_-exposed IL-10-null mice (an IBD model)^17^. In contrast, UFP-ingested atherosclerotic mice further developed a decreased abundance in Actinobacteria and Cyanobacteria, (Fig. 2D,E), whereas PM_10_-exposed IBD mice had no changes^17^. These differences in microbiota composition highlight the variations in PM sources, size and chemical compositions, as well as exposure duration, diet content, and animal models.

Gut microbiota influences numerous aspects of host energy and metabolism, including lipid metabolism^40^,^41^. A large portion of body cholesterol is synthesized in the intestine^42^, and conversion of cholesterol to bile acids is critical for maintaining cholesterol homeostasis and preventing accumulation of cholesterol, triglycerides, and toxic metabolites in the liver and other organs^43^. We previously showed that whole-body inhalation of UFP in *Ldlr*-null mice increased both intestinal and plasma lipid metabolites, including phospholipid lysophosphatidic acid (LPA), to promote atherosclerosis^4^,^5^. In the present study, UFP ingestion elevated pro-inflammatory lipid metabolites, including LPC, LPA, PEIPC, POVPC and PGPC (Fig. 4 and Supplemental Table S2). UFP ingestion also increased both intestinal and plasma LPC18:1 (Fig. 4D–G). Unsaturated LPC such as LPC18:1 is converted to inflammatory and atherogenic LPA by autotoxin to promote atherosclerosis^33^. Chattopadhyay *et al*. recently reported that *Ldlr*-null mice fed with LPC18:1 developed increased intestinal and systemic inflammation^44^, implicating LPC18:1 in UFP-mediated atherogenic responses.

At the family level, UFP ingestion decreased the relative abundance of Lachnospiraceae and a few species in the family (Supplemental Figure S1D–F) that are positively associated with diabetes, IBD, cirrhosis and prostate cancer^45^,^46^,^47^,^48^,^49^, but are negatively associated with colorectal cancer^35^,^36^,^37^. Intestinal inflammation promotes tumorigenesis by altering microbial composition and by inducing the expansion of microorganisms with genotoxic capabilities^50^.

Interestingly, Spearman’s analyses revealed that Actinobacteria was negatively correlated with intestinal and plasma LPC18:1 levels and cecal cholesterol levels (Fig. 6). These findings were in agreement with a recent report that decreased abundance in Actinobacteria was associated with increased cecal cholesterol excretion^40^. Furthermore, decreased abundance of cecal Cyanobacteria and Firmicutes was correlated with increased plasma LPC18:1 (Fig. 6E,F)^51^. This correlation is consistent with the relationship between obesity and the ecology of microbiota enriched in Actinobacteria, Cyanobacteria, and Firmicutes^18^,^52^,^53^,^54^. Taken together, our findings support the notion that UFP ingestion reduced Actinobacteria, Cyanobacteria, and Firmicutes to increase atherogenic lipid metabolites.

Alterations in gut permeability disrupt intestinal immune homeostasis^55^. PM ingestion increased gut permeability as assessed using FD4 or lactulose/mannitol gavage^17^,^56^. Analogous to UFP inhalation^4^,^5^, a moderate dose of UFP (PM_0.1_) ingestion at 40 μg/mouse/day, 3 days a week for 10 weeks, induced both intestinal and vascular pro-inflammatory mediators in the *Ldlr*-null mice (Figs 3 and 4). In addition, short-term PM (1 to 14 days) exposure was reported to increase intestinal inflammatory markers in association with increased gut permeability^17^,^52^ whereas our long-term exposure (10 weeks, 3 times/week) did not significantly alter gut permeability (*in vivo* and *ex vivo*) (Supplemental Figure S4A,B,C). *In vitro*, we observed that the colonic epithelial cell line, Caco-2, developed a dose-dependent increase in permeability to Straptavidin-HRP (97 kDa) at both 25 and 50 μg/ml of UFP treatment that is consistent with the findings of short term *in vivo* exposure (Supplemental Figure S4D). UFP ingestion increased *Akkermansia muciniphila*, the dominant *Verrucomicrobia* (Supplemental Figure S1C) that adheres to enterocytes and strengthens the integrity of the intestinal epithelium^39^. This may counterbalance the short-term increased permeability induced by UFP. Thus, the duration of UFP ingestion may influence gut epithelial permeability in the setting of altered microbiota composition.

For future studies, we would consider varying the UFP dose to recapitulate the range of human exposure. Given that gut permeability appears to be dependent on the duration of PM exposure, we are also interested in comparing acute versus chronic exposure to elucidate the possible protective effect of *Akkermansia*^39^. Based on our previous findings using an atherosclerosis model^4^,^5^, we elected to use *Ldlr*-null mice on a high fat diet in this study to demonstrate UFP-mediated alteration of the gut microbiota. In follow-up studies we will consider comparing the effects of UFP exposure in the setting of a high-fat or normal chow diet to determine how PM interacts with diet to shape the microbiome. The use of germ-free and antibiotic-treated mice would further elucidate the transmissibility through the microbiome of the intestinal and vascular phenotypes of UFP exposure.

Gut-vascular transmissibility is an emerging mechanism underlying the risk factor-mediated cardiovascular diseases. UFP ingestion by *Ldlr*-null mice induced decreased abundance of Actinobacteria, Cyanobacteria, and Firmicutes that correlated with the increased level of LPC18:1. Thus, we provide new gut-vascular insights into how PM affects microbiota composition and atherogenic mediators.

## Methods

### Ethics statement

All animal experiments were performed in compliance with UCLA Institutional Animal Care and Use Committee (IACUC) protocols, under a project license also approved by the UCLA IACUC. Humane care and use of animals were observed to minimize distress and discomfort.

### Collection and chemical analysis of UFP

Ultrafine particles (UFP, defined as particles with an aerodynamic diameter less than 0.8 μm) were collected on 8″ × 10″ Teflon filters using a high-volume ultrafine particle (HVUP) sampler^57^ at flow rate of 400 L/min, about 150 meters downwind of the I-110 Freeway in central Los Angeles, during January and February of 2015. The UFP represent a mixture of pollution sources, including fresh ambient PM from areas impacted by heavy-duty diesel trucks, light duty gasoline vehicles and ship emissions, as well as PM generated by photochemical oxidation of primary organic vapors^58^. The collection and characterization of chemical composition was previously described^5^. Mass fraction of the measured chemical species (in units of ng/μg UFP), including organic matter (estimated as total organic carbon content based on the method by Turpin *et al*.)^59^ and individual elements and metals, are reported in Supplemental Table S1. The most abundant elements in our samples included Ca (29.4 ng/μg PM), Na (23.2 ng/μg PM), S (22.6 ng/μg PM), Al (11.3 ng/μg PM) and Fe (10.0 ng/μg PM). As shown in Table S1, organic matter and metals/trace elements had cumulative mass fractions of 197 ng/μg PM and 116 ng/μg PM (i.e. about 20% and 12% of the UFP mass, respectively). The remaining UFP mass is mostly comprised of inorganic ions (most importantly nitrate, sulfate and ammonium)^60^,^61^. While these ions constitute a large fraction of the UFP mass, toxic properties of UFPs in the Los Angeles basin as well as most other urban areas of the world is primarily driven by the organic compounds and redox active metal species^62^ and therefore the chemical analyses in this study were focused on these UFP fractions.

### Mouse exposure to gavaged UFP

Age and weight-matched male low density lipoprotein receptor-null mice (*Ldlr*^–/–^) (at the age of 90 days and an average weight of 24.76 ± 0.37 g) on a C57BL/6 background (stock #002207, Jackson Laboratory, FA) were grouped randomly, and were orally administered with either vehicle control (11 mice) or 40 μg ambient UFP (12 mice) in 100 μL saline 3 days per week for 10 weeks via 22 gauge gavage needles. The orally administrated dosage of UFP at about 1.6 ug/g per session was determined based on our previous inhalation exposure at 400 μg/m^3^ assuming humans inhale 16 m^3^ air per day^63^. This dosage is about 5–10 folder lower than reported studies by Mutlu *et al*. and Kish *et al*.^17^,^56^. The mice were fed a Western-type high-fat diet (TD88137, 21% milk fat, 0.2% cholesterol, Harlan Laboratory) throughout the exposure period.

### Measurement of plasma cytokines

Mice were euthanized by inhalation of isofluorane and cervical dislocation following completion of the 10 weeks exposure. Plasma was collected using plasma separators (BD Biosciences) as previously described^64^. Plasma levels of TNF-α, MCP-1 and IL-6 were analyzed by a Luminex assay (Millipore: MCYTOMAG-70K-04. Mouse Cytokine MAGNETIC Kit).

### Immunohistochemistry

Ileum segments were harvested, fixed in PBS/4% paraformaldehyde, and embedded in paraffin blocks. Macrophages and neutrophils were stained using F4/80 antibody (Invitrogen, diluted at 1:100) and antibody against Ly6G (Biolegend, diluted at 1:100), respectively^65^. Intensity of F4/80 and Ly6G staining was quantified by the NIH ImageJ software (http://imagej.nih.gov/ij/) and presented as staining to background ratio.

### Quantification of lipid metabolites

Approximately 0.2–0.6 ml of blood was drawn for plasma preparation using plasma separators (BD Biosciences). The small intestines were dissected and a portion of the ileum was cut out and rinsed with cold saline. The extraction of lipid contents and measurement of lipid metabolites in plasma and intestine extracts were performed as previously described^4^,^64^. Lysophsophotidylcholine (LPC), lysophosphatidic acid (LPA), and oxidized phospholipids including 1-palmitoyl-2-epoxyisoprostane E2-*sn*-glycero-3-phosphorylcholine (PEIPC), 1-palmitoyl-2-oxovaleroyl-*sn*-glycero-3-phosphorylcholine (POVPC), and 1-palmitoyl-2-glutaroyl-*sn*-glycero-3-phosphorylcholine (PGPC) were analyzed and quantified by liquid chromatography, electron spray ionization, and tandem mass spectrometry as described previously (LC-ESI-MS/MS)^4^,^33^,^64^.

### Analysis of cecal bile acids and sterols

Cecum content was suspended in methanol, vortexed for 10 min, centrifuged at 14,000 g for 10 min at room temperature, and the supernatant was collected. Silylation of sterols and bile acids was carried out simultaneously using supernatant. After silylation, samples were centrifuged to collect supernatants for gas chromatography (GC). Samples were injected into RTX-5 column (Restek corp. 30 m × 0.25 mm × 0.25 i.d.) and simultaneous quantification of sterols and bile acids was performed using GC (Agilent 7890A) coupled to a FID. Peak identification was based on comparison of retention times with commercial standards of 5α-cholestane (internal standard), 5β-cholanic acid (internal standard), cholesterol, coprostanol, cholestanol, cholic acid, deoxycholic acid, chenodeoxycholic acid and lithocholic acid.

### Analysis of gut microbes via MiSeq sequencing

Cecum contents were used for DNA extraction using a commercial extraction system (PowerSoil DNA isolation kit, MO Bio laboratories, Inc). The quality of the DNA samples was confirmed using a Bio-Rad Experion system (Bio-Rad Laboratories, CA, USA). The 16S rRNA gene V4 variable region PCR primers 530/926 with barcode on the forward primer were used in a 30 cycle PCR using the HotStarTaq Plus Master Mix Kit (Qiagen, USA) under the following conditions: 94 °C for 3 min, followed by 28 cycles of 94 °C for 30 s, 53 °C for 40 s and 72 °C for 1 min, after which a final elongation step at 72 °C for 5 min was performed. Following amplification, PCR products were checked in 2% agarose gel to determine the success of amplification and the relative intensity of bands. Sequencing was performed at MR DNA (www.mrdnalab.com, Shallowater, TX, USA) on a MiSeq following the manufacturer’s guidelines. Sequence data were processed using a proprietary analysis pipeline (MR DNA, Shallowater, TX, USA). Operational taxonomic units (OTUs) were defined by clustering at 3% divergence (97% similarity). Final OTUs were taxonomically classified using BLASTn against a curated Green Genes database^66^.

### Principle component analysis (PCA) of microbiota

The dimensionality of the interrelated variables exhibiting the bacteria abundance was reduced by PCA^67^. Due to huge abundance differences among the bacteria, natural logarithm value of the abundance was input to the PCA to form a matrix of the number of bacteria in the taxonomic classification and the number of test samples. After obtaining the Eigen values and the Eigen vectors, the test samples were re-plotted in the coordinates of constructed by the first two Eigen vectors. The control and UFP groups were separated in the first two principal components. The PCA was applied to different taxonomic classifications; namely, phylum, class, order, family, genus, and species, respectively.

### Statistical analysis

Data were expressed as mean ± SEM unless otherwise stated. Statistical analysis was done with Matlab or Graphpad Prism. Multiple comparisons were performed by one-way analysis of variance (ANOVA), and statistical significance for comparison between two groups was determined by student t-test or Wilcoxon rank-sum test when data was not normally distributed. The association of lipids and metabolites with gut microbes was assessed by Spearman’s rank correlation analysis among all animals in both control and UFP exposed groups. A *p*-value < 0.05 was considered statistically significant. Sample size for different assays may differ from original mouse numbers due to issues of sample preparation, capacity of device, or sample limitations, which were specified per individual assay.

## Additional Information

**How to cite this article**: Li, R. *et al*. Ambient Ultrafine Particle Ingestion Alters Gut Microbiota in Association with Increased Atherogenic Lipid Metabolites. *Sci. Rep.* **7**, 42906; doi: 10.1038/srep42906 (2017).

**Publisher's note:** Springer Nature remains neutral with regard to jurisdictional claims in published maps and institutional affiliations.

## Supplementary Material

Supplementary Information
