# Supplementary material for: Ambient Ultrafine Particle Ingestion Alters Gut Microbiota in Association with Increased Atherogenic Lipid Metabolites
Source: Sci Rep. 2017 Feb 17;7:42906. doi: 10.1038/srep42906 (PMC5314329; doi:10.1038/srep42906)
Supplement: Supplementary Information [file srep42906-s1.pdf]

Supplementary Information

Ambient Ultrafine Particle Ingestion Alters Gut Microbiota in Association with Increased Atherogenic Lipid Metabolites

Rongsong Li,[1] Jieping Yang,[2] Arian Saffari,[3] Jonathan Jacobs,[4] Kyung In Baek,[5] Greg Hough,[1] Muriel H. Larauche,[4] Jianguo Ma,[1,5] Nelson Jen,[1,5] Nabila Moussaoui,[4] Bill Zhou,[1] Hanul Kang,[1] Srinivasa Reddy,[1] Susanne M. Henning,[2] Matthew J. Campen[6], Joseph Pisegna[4], Zhaoping Li,[2] Alan M. Fogelman,[1] Constantinos Sioutas,[3] Mohamad Navab,[1] Tzung K. Hsiai[1,5]

[1]Division of Cardiology, Department of Medicine, School of Medicine, University of California, Los Angeles, CA 90095
[2]Division of Clinical Nutrition, Department of Medicine, School of Medicine, University of California, Los Angeles, CA 90095
[3]Civil and Environmental Engineering, University of Southern California, Los Angeles, CA 90089
[4]Division of Gastroenterology and Hepatology, Department of Medicine, School of Medicine, University of California, Los Angeles, CA 90095
[5]Department of Bioengineering, School of Engineering & Applied Science, University of California, Los Angeles, CA 90095
[6]Department of Pharmaceutical Sciences, School of Pharmacy, University of New Mexico, Albuquerque, NM 87131

Corresponding Author:
Tzung K. Hsiai, M.D., Ph.D.
Department of Medicine and Bioengineering
David Geffen School of Medicine
UCLA School of Engineering & Applied Science
11301 Wilshire Blvd. 111E
Los Angeles, CA 90089
Email: thsiai@mednet.ucla.edu
Phone: (310)268-3839
Fax: (310) 268-4288

Running Title: UFP, Gut Microbiota and Pro-inflammatory Mediators

Supplemental Methods:

16S ribosomal RNA sequence analysis

Raw MiSeq sequence data was processed in QIIME 1.9.1 using default quality parameters (Caporaso *et al.* 2010). Closed-reference operational taxonomic unit (OTU) picking at the 97% similarity threshold was performed in QIIME against the May 2013 version of the Greengenes database (http://greengenes.secondgenome.com), pre-filtered at 97% identity. Alpha and beta diversity were assessed using data rarefied to 32,346 sequences. Principal coordinates analysis (PCoA) was performed with distance matrices calculated using unweighted and weighted UniFrac (Lozupone *et al.* 2005). Adonis with 100,000 permutations was used to assess statistical significance of differences in beta diversity (Anderson 2001).

Differential abundance testing of OTUs, genera, or phyla was performed using phyloseq and the DESeq2 algorithm (http://www.bioconductor.org/packages/release/bioc/html/DESeq2.html) (McMurdie *et al.* 2013, Love *et al.* 2014). OTUs present in only a single sample were removed prior to analysis. An empirical Bayesian approach was used to shrink dispersion of normalized count data. Log fold changes for each OTU were fitted to a negative binomial model. Taxa were filtered out by choosing a mean count threshold maximizing the number of taxa returned at a given false discovery rate. Outliers were replaced by trimmed means. P-values were calculated using the Wald test then converted to q-values (http://www.bioconductor.org/packages/release/bioc/html/qvalue.html).

Gut permeability assay

In vivo intestinal permeability assay: The assay is essentially done as previously described (Moussaoui et al. 2014). Briefly, mice were given an oral gavage of fluorescein isothiocyanate (FITC) labeled 4-kD dextran (FD4, Sigma)) at the dose of 12 mg/ml in 0.9% NaCl. After 4 hours, mice were anesthetized with isoflurane and lightly hand restrained and a blood sample (200 µl) was withdrawn within a few seconds from the facial vein. Samples were placed immediately in heparin-coated tubes on ice and centrifuged. Intestinal permeability was determined by measuring plasmatic FD4 concentration using an automatic synergy HT plate reader (Ex 485 nm; Em 525 nm, BioTek).

Ex-vivo permeability assay: At the end of 10 week UFP exposure, pieces of ileum tissue from euthanized mice were mounted on 0.10 cm^2 slides dedicated to Ussing chamber. The intestinal paracellular permeability were assessed by measuring mucosal-to-serosal flux of FITC-labeled 4-kDa dextran (FD4; 2.2 mg/ml; Sigma) across the intestinal strip every 30 min for 2 hours as done previously with a slight modification (Moussaoui et al. 2014).

***In vitro* permeability assay**

Enterocytes CACO-2 were seeded into clear transwell (Corning Inc, 6.5mm diameter, 0.4µm pore size)at $1X10^5$ per well in 24-well plate. Cells were grown to complete confluence in 7-10 days with media change every 2 days. Cells were then rinsed with treatment media (M199/0.1%FBS) and treated with or without UFP in treatment media in the presence of 1µg/ml of Horse Radish Peroxidase-Streptavidin (HRP-Streptavidin, Thermofisher) in the top transwell.

One ml of treatment media was added to the bottom well. Changes in endothelial permeability were analyzed by the HRP activities of media in the bottom well.

For the HRP activity assay, five µl of media from bottom well were taken and mixed with 100 µl of TMB solution (HRP substrate, Thermofisher Inc) in wells of 96-well plate. After incubation for 5-30 minutes at a room temperature, one hundred µl of 2M sulfuric acid was added to stop the reaction. The absorbance at 450 nm (OD 450) was read as HRP activities.

References:

Anderson, M.J. 2001. A new method for non-parametric multivariate analysis of variance. Austral Ecology, 26: 32–46.

Caporaso JG, Kuczynski J, Stombaugh J, Bittinger K, Bushman FD, Costello EK, et al. QIIME allows analysis of high-throughput community sequencing data. Nat Methods. 2010;7(5):335-6.

Love MI, Huber W, Anders S. Moderated estimation of fold change and dispersion for RNA-seq data with DESeq2. Genome biology. 2014;15(12):550.

Lozupone C, Knight R. UniFrac: a new phylogenetic method for comparing microbial communities. Applied and environmental microbiology. 2005;71(12):8228-35.

McMurdie PJ, Holmes S. phyloseq: an R package for reproducible interactive analysis and graphics of microbiome census data. PloS one. 2013;8(4):e61217.

Moussaoui N, Braniste V, Ait-Belgnaoui A, Gabanou M, Sekkal S, Olier M, et al. 2014. Changes in intestinal glucocorticoid sensitivity in early life shape the risk of epithelial barrier defect in maternal-deprived rats. PLoS One 9(2): e88382.

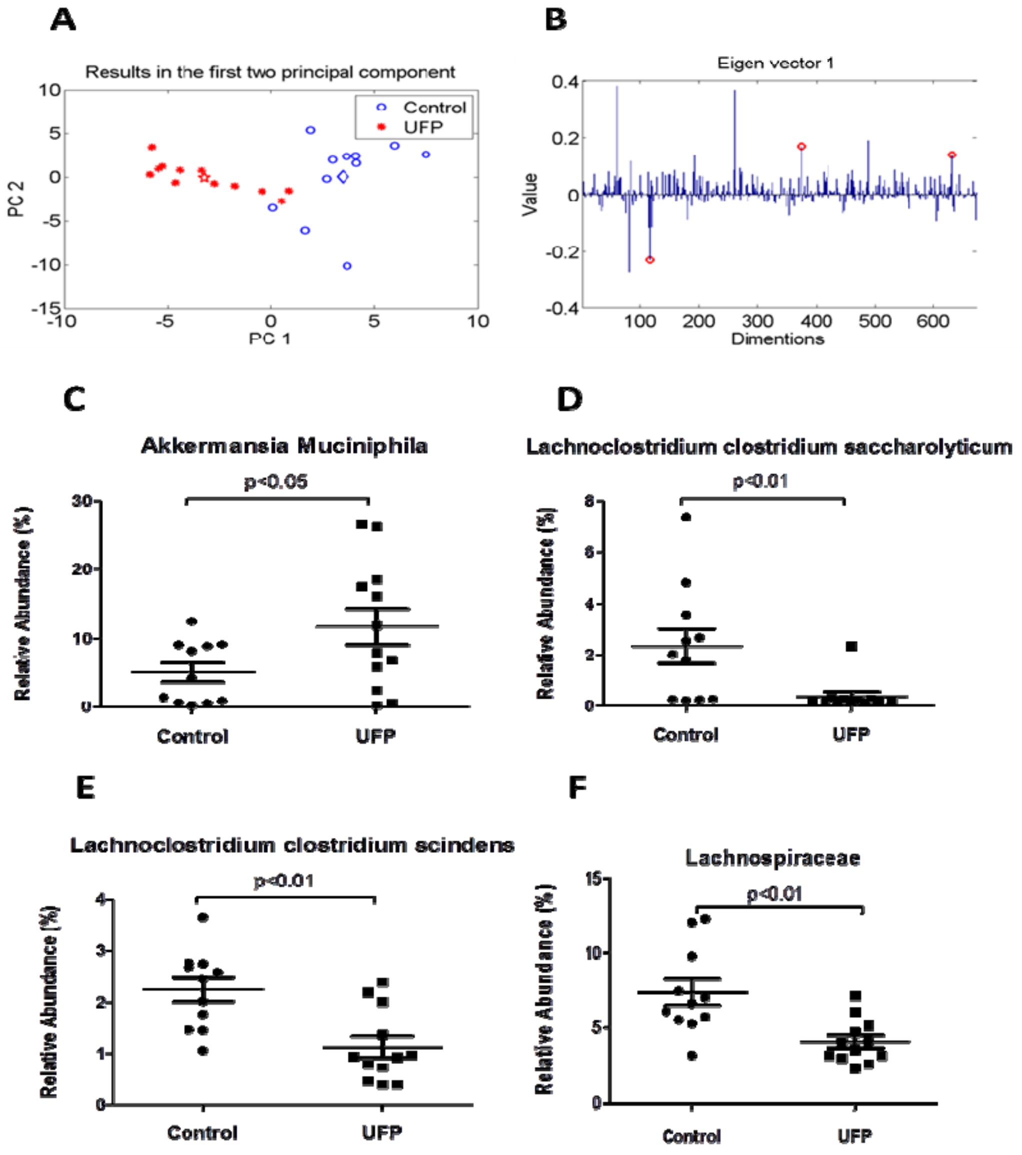

Supplemental Material Figure S1. UFP exposure altered the dominant species *Akkermansia muciniphila,* in the Verrucomicrobia phylum, and species in the Lachnospiraceae family. DNAs were isolated from the cecal contents and applied to MiSeq sequencing for microbiota analysis. The relative abundance of bacteria was calculated based on operational taxonomic units (OTUs). (**A**) PCA at the OTU level (roughly corresponding to species) revealed segregation of microbe abundance between control and UFP exposure. The mean UFP value (red pentagram) was significantly lower than that of the control (blue diamond) in the first principal component (PC1, $p < 0.0001$). (**B**) The first Eigen vector revealed OTUs (peaks) that were different between control group and UFP exposure group. The three red circles indicate *Akkermansia muciniphila, Lachnoclostridium Clostridium saccharolyticum* and *Lachnoclostridium Clostridium scindens*. (**C**) Relative abundance of *Akkermansia muciniphila.* (**D**) Relative abundance of *Lachnoclostrium Clostridium saccharolyticum.* (**E**) Relative abundance of *Lachnoclostrium Clostridium scindens.* (**F**) Relative abundance of the Lachnospiraceae family. (n=11-12)

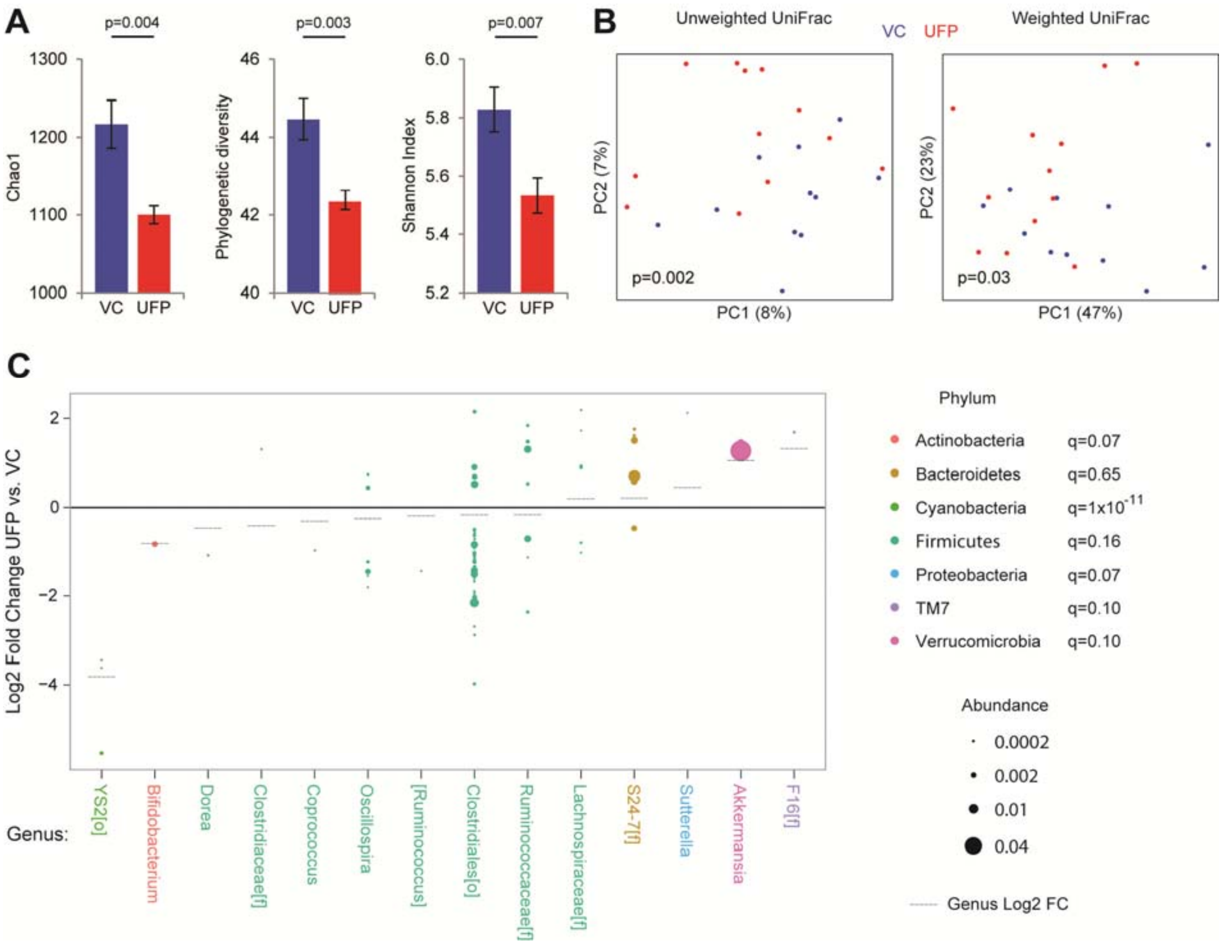

Supplemental Material Figure S2. UFP ingestion reduced microbial diversity compared to vehicle control (VC) in *Ldlr*-null mice, associated with expansion of *Akkermansia muciniphila* and depletion of microbes within the Cyanobacteria, Actinobacteria, and Firmicutes phyla. (**A**) Alpha diversity is compared between the UFP and VC groups using three metrics: Chao1 (richness), Faith's phylogenetic diversity, and Shannon index (evenness). P-values were calculated using two-tailed t-tests. (**B**) Principal Coordinates Analysis plots are shown to visualize differences in microbial composition across samples (beta diversity) as assessed by unweighted and weighted UniFrac. Each dot represents a mouse, colored by exposure status. P-values were calculated using Adonis. (**C**) OTUs with a statistically significant difference in abundance between the UFP and VC groups are shown. Effect size is represented as the log2 fold change (FC). The size of each dot is proportional to the normalized abundance of that OTU. The genus of each OTU is shown on the horizontal axis. Genera are ordered by the log2 FC between the UFP and VC groups of the genus as a whole. Families [f] or orders [o] are indicated for OTUs that did not have an assigned genus in the Greengenes database. Color signifies phylum. Q-values are shown for the significance of phylum level changes in abundance.

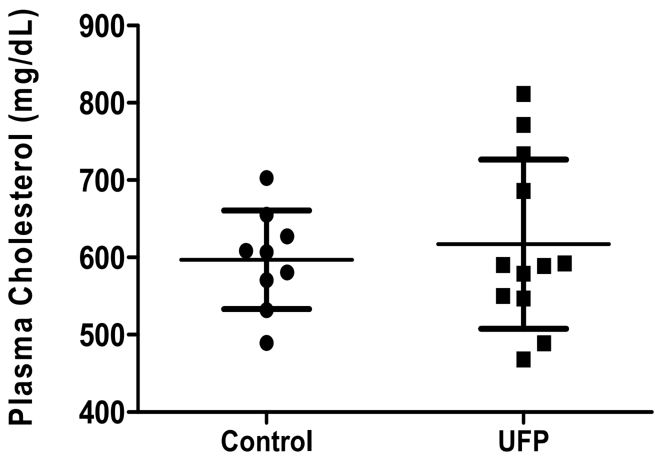

Supplemental Material Figure S3. UFP exposure via oral gavage did not alter plasma levels of cholesterol. The plasma levels of total cholesterol from mice exposed to vehicle control or UFP were measured with a colorimetric assay kit from Pointe Scientific. There was no significant change of plasma cholesterol.

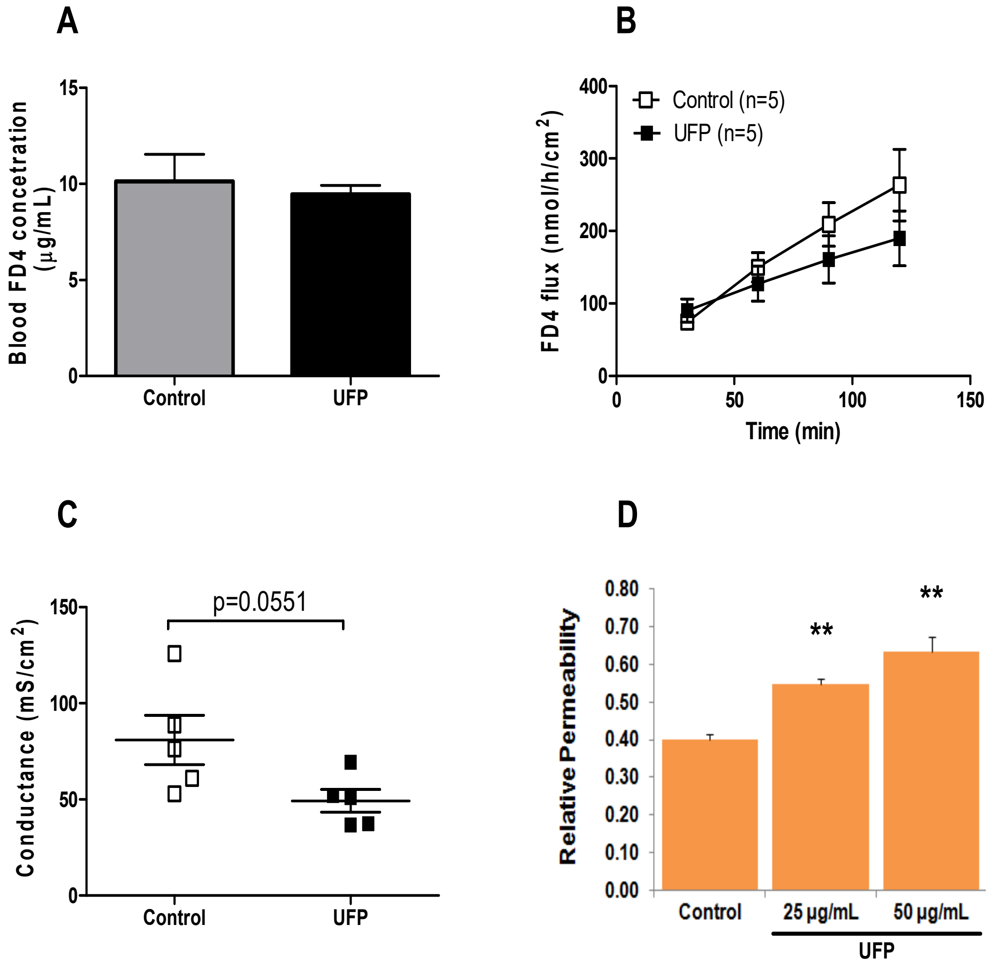

Supplemental Material Figure S4. Effect of gavaged UFP on gut permeability. (**A**) *In vivo* gut permeability assay was conducted in mice exposed to vehicle control or UFP at week 9 as described in the methods (n=11). (**B**) *Ex vivo* ileal permeability was assessed by measuring mucosal-to-serosal flux of FITC-labeled 4kDa dextran (**FD4**) (n=5). (**C**) Conductance measurement of ileal tissue mounted on Ussing chamber (n=5). **(D)** A confluent monolayer of CACO-2 cells was treated with UFP for 6 hours in the presence of 1μg/ml of Streptavidin-HRP for relative permeability measurement. UFP increased the permeability of CACO-2 cells (** $p < 0.001$ vs. control, n=3) with a trend towards a dose response that fell short of significance ($p > 0.05$).

Supplemental Material Table S1. Mass fraction of major chemical species (including metals and trace elements, in addition to organic matter). Errors correspond to standard deviations of 3 samples.

Species	Concentration (ng/µg UFP)	Species	Concentration (ng/µg UFP)
Organic Matter	197 (±22)	Ce	0.011 (±0.004)
Ca	29.4 (±10.3)	As	0.011 (±0.004)
Na	23.2 (±8.43)	Co	0.009 (±0.003)
S	22.6 (±7.46)	La	0.005 (±0.001)
Al	11.3 (±3.67)	W	0.005 (±0.001)
Fe	10.0 (±3.06)	Nd	0.003 (±0.001)
Mg	6.85 (±2.29)	Y	0.003 (±0.001)
K	6.53 (±2.10)	Cd	0.002 (±0.0007)
Zn	1.86 (±0.618)	Ag	0.001 (±0.0007)
P	0.630 (±0.192)	Sc	0.002 (±0.0004)
Ba	0.612 (±0.222)	Nb	0.001 (±0.0004)
B	0.575 (±0.453)	Pd	0.001 (±0.0004)
Ti	0.545 (±0.171)	Th	0.001 (±0.0003)
Cu	0.499 (±0.156)	Pr	0.001 (±0.0003)
Sr	0.26 (±0.086)	Cs	0.0007 (±0.0002)
Mn	0.231 (±0.072)	Sm	0.0006 (±0.0001)
Pb	0.165 (±0.065)	U	0.0005 (±0.0001)
Ni	0.156 (±0.050)	Dy	0.0004 (±0.0001)
Cr	0.153 (±0.051)	Pt	0.0003 (±0.0003)
Sb	0.093 (±0.031)	Tl	0.0003 (±0.0001)
Sn	0.079 (±0.024)	Eu	0.0002 (±0.0001)
Mo	0.043 (±0.014)	Yb	0.0002 (±0.00008)
V	0.028 (±0.009)	Ho	0.00009 (±0.00002)
Li	0.019 (±0.006)	Rh	0.00008 (±0.00003)
Rb	0.016 (±0.005)	Lu	0.00003 (±0.00001)

Supplemental Material Table S2: Effects of UFP exposure via oral gavage on phospholipids

Tissue	LPA 18:1	LPA 18:2	LPA 20:4	PEIPC	POVPC	PGPC
**Intestine (ng/gram tissue)**						
Control	1972±101.4	3730 ± 407.6	2155 ± 168.4	12.34 ± 0.94	0.063 ± 0.005	0.054 ± 0.007
UFP	5207 ± 461.3 ***	6881 ± 765.4 **	4967 ± 576.7***	42.69±5.49***	0.189 ± 0.017***	0.145 ± 0.014***
**Plasma (ng/mL)**						
Control	88.3 ± 24.0	185.0± 25.0	95.7 ± 24.1	1276 ±116.3	25.5 ±3.9	26.1 ± 3.4
UFP	175.9 ± 28.1 *	324.6 ± 54.4 *	171.1 ± 19.9*	1960 ± 157.2**	46.3 ± 5.0**	47.7 ± 6.1**

Note: Values are means ± SEMs; UFP vs. Control, * p<0.05 ; **p<0.01; *** p<0.001

Supplemental Material Table S3: Effects of UFP exposure via oral gavage on cecal bile acids

ug/g cecal contents	**VC**	**UFP**
**Lithocholic acid**	72.7 ± 4.9	73.4 ± 5.9
**Deoxycholic acid**	428.5 ± 84.2	536.1 ± 72.5
**Cholic acid**	129.9 ± 33.1	163.5 ± 60.0
**Chenodeoxycholic acid**	12.0 ± 2.7	16.1 ± 5.1
**Total bile acids**	643.2 ± 99.7	789.0 ± 132.0
**Lith/Deoxy**	0.2 ± 0.0	0.2 ± 0.0

Note: Values are means ± SEMs (n = 11-12).
